# Study on the Joint Toxicity of BPZ, BPS, BPC and BPF to Zebrafish

**DOI:** 10.3390/molecules26144180

**Published:** 2021-07-09

**Authors:** Ying Han, Yumeng Fei, Mingxin Wang, Yingang Xue, Hui Chen, Yuxuan Liu

**Affiliations:** 1 School of Environmental & Safety Engineering, Changzhou University, Changzhou 213164, China; feiyumeng1997@163.com (Y.F.); yzxyg@126.com (Y.X.); huichen@cczu.edu.cn (H.C.); dinoice0401@163.com (Y.L.); 2 Jiangsu Engineering Research Center of Petrochemical Safety and Environmental Protection, Changzhou 213164, China

**Keywords:** bisphenol analogues, zebrafish, joint toxicity, gene expression

## Abstract

Bisphenol Z (BPZ), bisphenol S (BPS), bisphenol C (BPC), and bisphenol F (BPF) had been widely used as alternatives to bisphenol A (BPA), but the toxicity data of these bisphenol analogues were very limited. In this study, the joint toxicity of BPZ, BPS, BPC, and BPF to zebrafish (*Danio rerio*) was investigated. The median half lethal concentrations (LC50) of BPZ, BPS, BPC, and BPF to zebrafish for 96 h were 6.9 × 10^5^ µM, 3.9 × 10^7^ µM, 7.1 × 10^5^ µM, and1.6 × 10^6^ µM, respectively. The joint toxicity effect of BPF–BPC (7.7 × 10^5^–3.4 × 10^5^µM) and BPZ–BPC (3.4 × 10^5^–3.5 × 10^5^µM) with the same toxic ratio showed a synergistic effect, which may be attributed to enzyme inhibition or induction theory. While the toxicity effect of the other two bisphenol analogue combined groups and multi-joint pairs showed an antagonistic effect due to the competition site, other causes need to be further explored. Meanwhile, the expression levels of the estrogen receptor genes (ERα, ERβ1) and antioxidant enzyme genes (SOD, CAT, GPX) were analyzed using a quantitative real-time polymerase chain reaction in zebrafish exposure to LC_50_ of BPZ, BPS, BPC, and BPF collected at 24, 48, 72, and 96 h. Relative expression of CAT, GPX, and ERβ1 mRNA declined significantly compared to the blank control, which might be a major cause of oxidant injury of antioxidant systems and the disruption of the endocrine systems in zebrafish.

## 1. Introduction

Bisphenol analogues are typical environmental endocrine disruptors and are prone to accumulate in water bodies. With the limitation of bisphenol A (BPA), the production and application of BPA substitutes such as such as bisphenol S (BPS), bisphenol F (BPF), bisphenol C (BPC) and bisphenol Z (BPZ) has gradually increased, and these substitutes release large amounts of bisphenol analogues into the water, increasing the safety risk of the growth and development of fish-based aquatic organisms [1,2]. BPF can be used instead of BPA to make epoxy resin. BPS is mainly used as a color fixing agent, as a resin flame retardant, and as materials in color photography. BPC is often used in the preparation of flame retardant. BPZ can be applied in the manufacturing of chemical compounds [3]. The estrogen activities and octyl alcohol water distribution coefficients of BPZ, BPS, BPC, and BPF are similar to those of BPA or may be even higher, which may pose risks to aquatic ecosystems and human health [4].

Bisphenol analogues have been found in different media [5]. Liao et al. collected indoor dust samples from the United States, China, Japan, and South Korea. The total content of eight bisphenols in the dust ranged from 0.026 to 111 μg·g^−1^ (mean 2.3 μg·g^−1^). BPA, BPS, and BPF were the three main bisphenols, accounting for 98% of the total concentrations [6]. The content of seven bisphenol analogues in surface water and sediments from Lake Taihu (range: 81–3.0 × 10^3^ ng·L^−1^) was higher than that of Lake Luoma (range: 1.5 × 10^2^–1.9 × 10^3^ ng·L^−1^) [7]. Chen et al. determined the concentrations of 7 kinds of bisphenol analogues in the urine of 283 samples of children aged 3–11 years in South China and found that the total concentrations of 7 bisphenol analogues in urine ranged from 0.43 μg·L^−1^ to 32 μg·L^−1^ [8]. In addition, Kha et al. also investigated the genotoxicity of BPA, BPS, BPF, BPAF, and their combinations [9]. Mu et al. conducted an acute toxicity test of four bisphenol analogues on zebrafish embryos, in which the half lethal concentration (LC_50_) value of BPF for 96 h was 3.9 × 10^6^ µM [10].

Zebrafish (*Danio rerio*), a type of small tropical fish, has organs and systems that were initially used for neurodevelopmental and genetic related research. As a new type of vertebrate model organism, zebrafish have gradually entered into more fields [11,12]. Currently, studies on the toxic effects of bisphenol analogues on zebrafish mainly focus on single toxicity and mechanisms. Zhao et al. exposed male zebrafish to 2.5 × 10^2^ µM and 2.5 × 10^3^ µM BPS solutions for 28 days and found that the plasma insulin level of male zebrafish was significantly reduced, thus impeding its physiological effect on glucose metabolism, leading to increased liver glucose output and decreased glucose metabolism and storage [13]. Sangwoo et al. studied the interfering effects of BPF, BPS, and BPZ on the thyroid hormone in zebrafish embryo larva, finding that they can damage the thyroid function in juvenile fish, and their damaging effects may be greater than that of BPA [14]. The exposure of zebrafish embryos to different concentrations of BPS (0, 0.1, 1, 10, and 100 μg·L^−1^) for 75 days was performed and adverse effects on the endocrine system such as decreasing gonadosomatic index, decreasing plasma 17β-estradiol, and increasing hepatosomatic index were observed [15]. Ji et al. discovered significantly decreasing concentrations of testosterone with the *cyp19a* upregulated gene and the *cyp17* and *17βhsd* downregulated genes [16] Niederberger et al. showed that exposure to 10 µM of BPA, BPS, and BPF reduced the expression of *Insl3* in tests with cultured mouse fetuses [17]. In addition to BPS and BPF, other BPs were also detected at very low concentrations. However, the enrichment effect and acute toxicity of these BPs were higher than those of BPS and BPF [18].

In this study, we investigated 96-h single and joint acute toxicity experiments of BPZ, BPS, BPC, and BPF on zebrafish and evaluated the expression of key genes that vary with the exposure of BPZ, BPS, BPC, and BPF. The aim of the study was to identify the what impacts emitted bisphenol analogues other than BPA may have on zebrafish and to assess the impacts of these bisphenol analogues on aquatic ecosystems.

## 2. Results and Discussion

### 2.1. Single Acute Toxicity of BPZ, BPC, BPF and BPS to Zebrafish

The structures and physicochemical properties of BPZ, BPS, BPC, and BPF are shown in Table 1. The increasing molecular weight ranking order was BPF, BPS, BPC, and BPZ. The octanol-water partition coefficient (Log Kow) of BPS, BPF, BPC, and BPZ had similar trends to their bioaccumulation factors (Log BAF). The Log Kow value of BPZ was the highest, followed by that of BPC, BPF, and BPS. Stronger Log Kow values of the bisphenol analogues implied a stronger biological enrichment ability.

**Figure 1:**
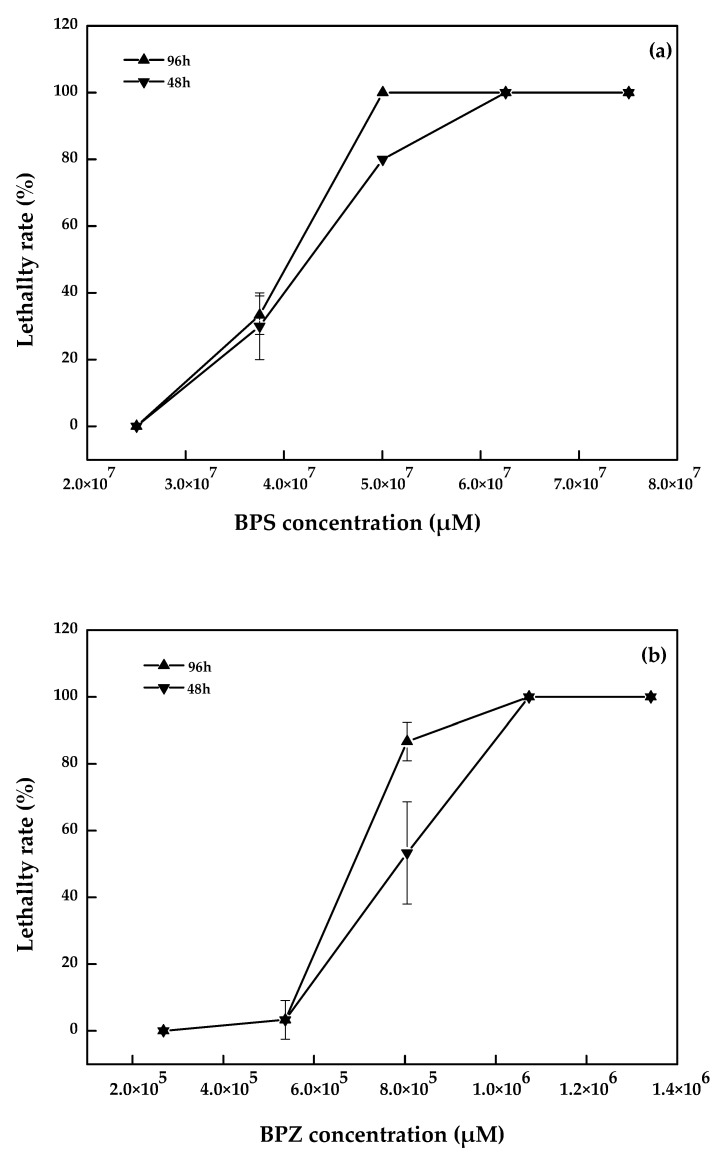

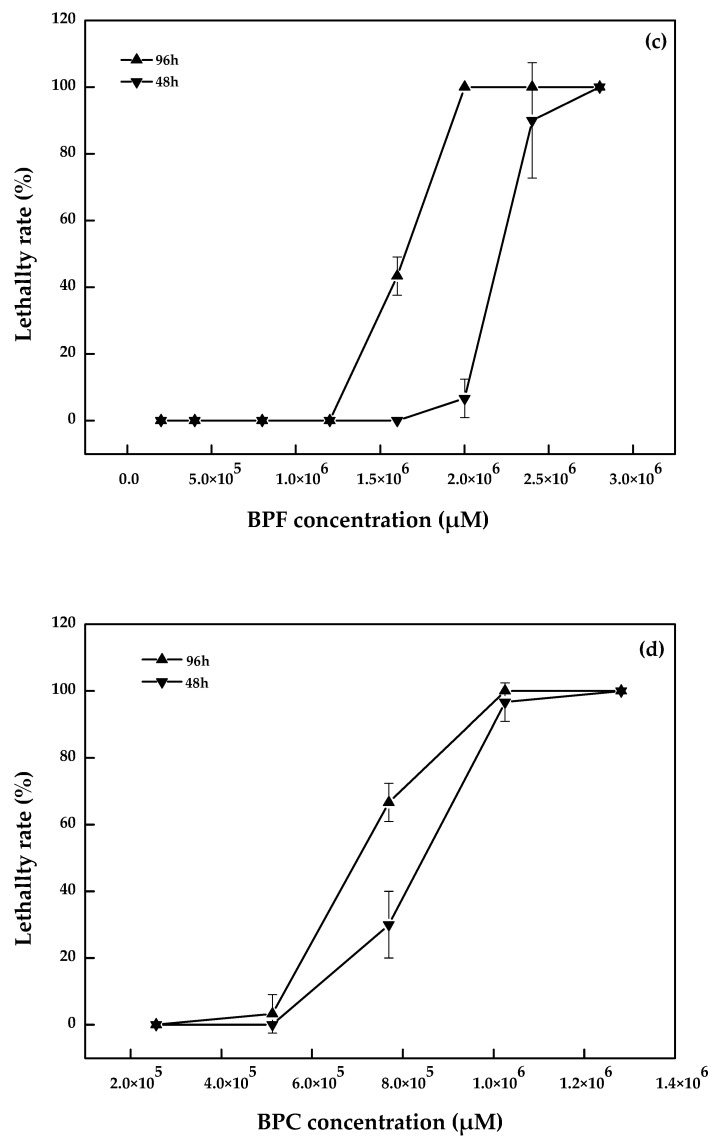
BPS (**a**), BPZ (**b**), BPF (**c**), and BPC (**d**) exposure on zebrafish after 48 h and 96 h (values represent mean ± S.D., sample size: 3).

No death or abnormality in the zebrafish in the blank and solvent control groups were observed. The linear regression analysis results of toxicity test data for zebrafish were shown in Figure 1. The LC_50_ of BPZ, BPC, BPF, and BPS was 6.9 × 10^5^ µM, 7.1 × 10^5^ µM, 1.6 × 10^6^ µM, and 3.9 × 10^7^ µM, respectively. The toxicity of these four bisphenol analogues was BPZ > BPC > BPF > BPS. According to relevant studies, the acute toxicity of the compounds to the fish was divided into 5 grades: LC_50_ < 1 mg·L^−1^ was extremely hazardous, 1 ≤ LC_50_ < 100 mg·L^−1^ was highly hazardous, 100 ≤ LC_50_ < 1000 mg·L^−1^ was moderately hazardous, 1000 ≤ LC_50_ < 10,000 mg·L^−1^ was slightly hazardous, and LC_50_ ≥ 10,000 mg·L^−1^ was mildly toxic [19]. Therefore, the toxicity of BPF, BPZ, and BPC belong to the highly hazardous group, while BPS was considered to be moderately hazardous.

Ren et al. chose adult zebrafish and embryos as test organisms and did not feed or change the water during their experiment. The LC_50_ values of BPZ in adult zebrafish and embryos after 96 h were 7.1 × 10^5^ µM and 7.9 × 10^5^ µM, respectively. For BPF and BPS, the LC_50_ values in adult zebrafish were 1.9 × 10^6^ µM and 8.6 × 10^7^ µM, and in embryo, they were 1.5 × 10^6^ µM and 8.1 × 10^7^ µM [20]. Relevant research on BPC was limited. The toxicity of BPZ, BPF, and BPS was BPZ > BPF > BPS, which was consistent with this study. The toxicity of BPS was two orders of magnitude smaller than the other three bisphenol analogues. The numerical differences between the results may be related to the growth environment, the biological state of test organism, and the experimental conditions.

### 2.2. Dual Joint Acute Toxicity of BPZ, BPC, BPF and BPS to Zebrafish

**Figure 2:**
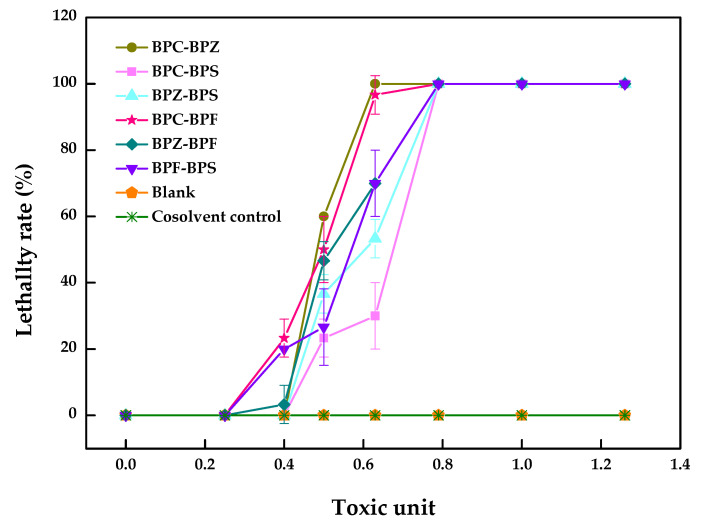
Combined toxicity of BPZ, BPC, BPF, and BPS on zebrafish after 96 h (values represent mean ± S.D., sample size: 3).

The pairwise LC_50_ values of BPZ, BPC, BPF, and BPS with equal toxicity ratios to zebrafish after 96 h and the dual combined effect of these four compounds were calculated and evaluated. As shown in Table 2, the dual combined effect of BPF–BPC (3.4 × 10^5^–7.7 × 10^5^ µM) with an additive index (AI) value of 0.05 and BPZ–BPC (3.4 × 10^5^–3.5 × 10^5^ µM) with an AI value of 0.01 showed synergistic effect, while the rest of the groups with negative AI values showed an antagonistic effect, which can also be seen from Figure 2.

As shown in Figure 2, a clear trend was not observed for the decrease in the percentage for the lethality rate in BPF–BPC and BPZ–BPC. However, there was sudden drop in the performance of another four joint groups, meaning that the antagonistic effect may exist. A small number of studies were carried out to explore the combined toxic effects of BPZ, BPC, BPF and BPS to zebrafish, which may promote the inhibition or induction of relative genes expression. Enzyme inhibition or induction theory states that one compound converts to the enzyme inducer of another one in a hybrid system, which may promote the detoxification or activation the toxicity of another compound, presenting a synergy [21]. The mechanisms of the combined toxic effects need to be further identified.

Lei et al. reported that the joint toxicity of BPA and nitrophenol on loach as an antagonism effect was due to the nitrophenol reactive ion occupied in the limited binding sites in the cells, reducing the combination efficiency of the BPA reactive ion [22]. Fang et al. found three cresol isomers with similar chemical properties appearing to interfere during a short period of time and reduce the toxicity to the zebrafish [23]. BPZ, BPC, BPF, and BPS had two hydroxyl phenyl groups with different substituents on the carbon bridge. The antagonistic effect may be caused by the competing effects of bisphenol analogues on the active sites of the cell surface and the metabolic system. One substance occupied the binding site on the cell surface and reduced the binding of other substances. The toxicity was inhibited and resulted in an overall antagonistic effect.

### 2.3. Multi-Joint Acute Toxicity of BPZ, BPC, BPF and BPS to Zebrafish

**Figure 3:**
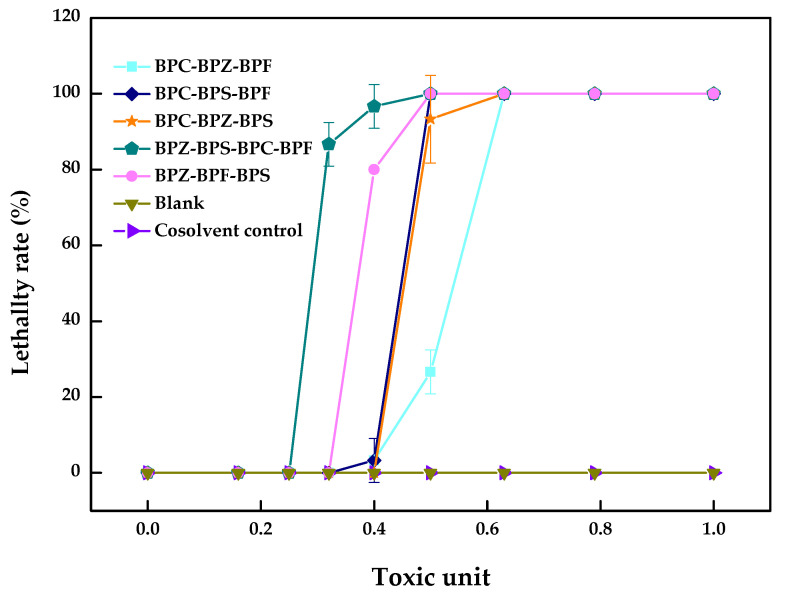
Multi-joint acute toxicity of BPZ, BPC, BPF, and BPS on zebrafish after 96 h (values represent mean ± S.D., sample size: 3).

It can be seen from Table 3 and Figure 3 that the AI values of the multi-joint acute toxicity of BPZ, BPC, BPF, and BPS with equal toxic ratios to zebrafish were all negative, which showed an antagonistic effect and is consistent with most conclusions obtained from dual combined acute toxicity test.

The mechanism and function of each component in the activation site differences leads to the different joint toxicity effects. Each component’s contribution to the ultimate toxic effects was different, especially for mixtures containing more than three components. The appearance of an antagonistic effect meant that the decisive composition in the zebrafish had an inconsistent toxicity effect or competition site. In addition to the above reasons, different exposure times and matching methods would also change the determination of joint toxicity. At present, data on the toxicity of the combined action of bisphenol analogues are limited even though the mechanism of the biological toxicity of bisphenol analogues is multi-faceted and very complex. The specific mechanism needs to be further studied and discussed.

### 2.4. Effects of BPZ, BPC, BPF and BPS Exposure on Relevant Gene Expression

Bisphenol analogues have interfering effects on both endogenous hormones determined by the expression of catalase (CAT), peroxide dismutase (SOD) and glutamine peroxidase (GPX) as well as the anti-oxidation system that is influenced by the expression of estrogen receptor (ERα and ERβ1) [24,25,26]. Consequently, the determination of CAT, SOD, GPX, ERα and ERβ1 expression during exposure to LC_50_ of BPC, BPZ, BPF, and BPS on zebrafish was investigated on the basis of the reference gene ribosomal protein 17 (*rp17*). The blank sample was set as control. The threshold cycle (Ct) was used to evaluate the variations. Previous studies focused on relationships between gene expression and different concentrations of chemical exposure groups. For instance, Xu et al. investigated the immunotoxicity of dibutyl phthalate to zebrafish, which upregulated the expression of *rag1/2* [27]. In the present study, concentration-dependent groups were not created, but there were different exposure time-dependent groups instead, which were limited. Based on our results, the differences in the relative expression of both SOD and Erα was nonsignificant, and the relative gene expression of CAT, GPX, and ERβ1 was significant.

**Figure 4:**
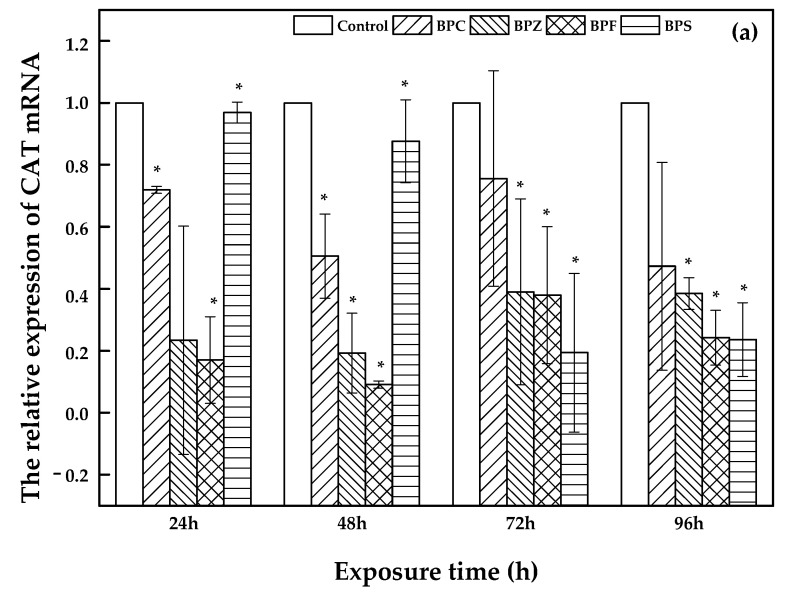

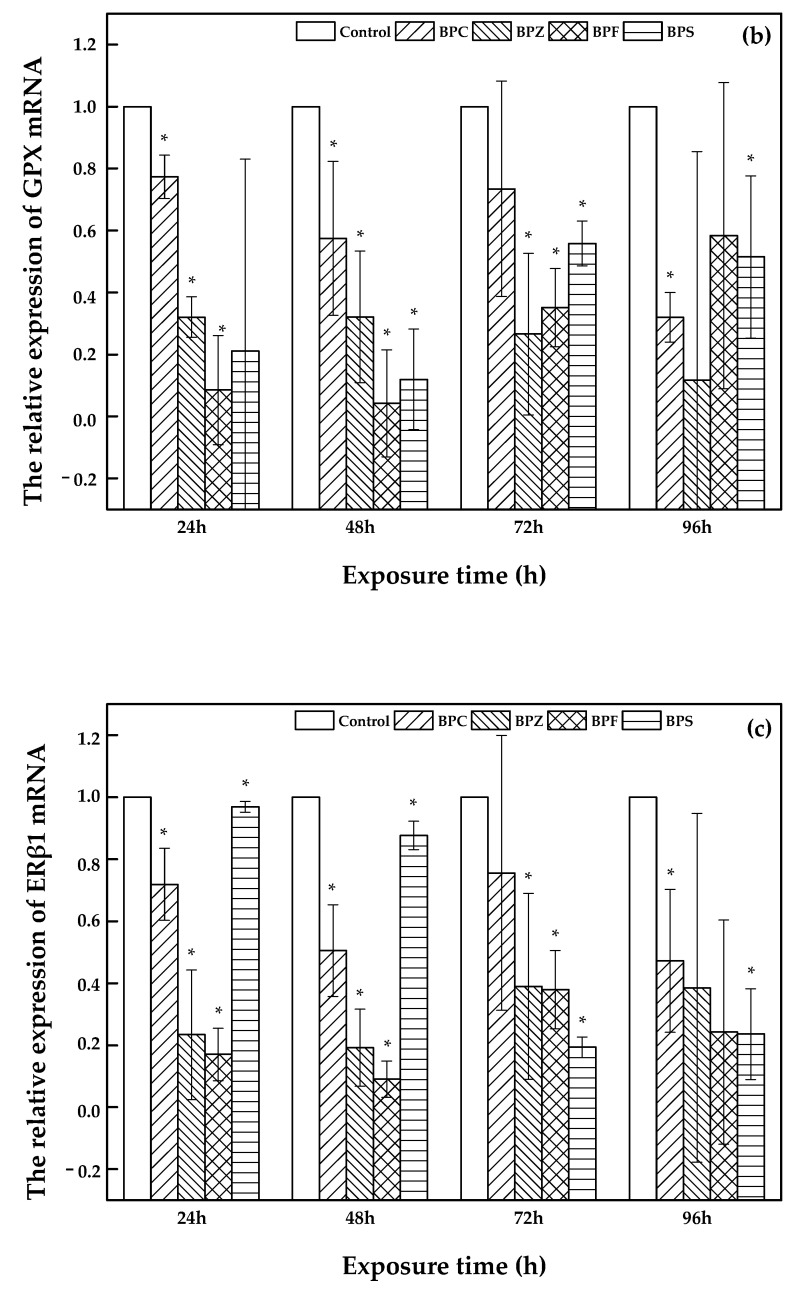
Relative expression of CAT (**a**), GPX (**b**), and ERβ1 (**c**) in treated and control groups (values represent mean ± S.D.; sample size: 3; * significant difference compared to the blank control (*p* < 0.05)).

As shown in Figure 4, a description of CAT, GPX, and ERβ1 expression was illustrated. Bars represent the relative change in mRNA expression in the treated group compared to the control group. With an increasing exposure time (24 h, 48 h, 72 h, and 96 h) of zebrafish to BPC, BPZ, BPF, and BPS, there was a fluctuating relative expression of CAT, GPX, and ERβ1. However, compared to the control group, relative expression of CAT, GPX, and ERβ1 was significantly decreased in all of the treated groups. Salahinejad et al. evaluated the expression of CAT in zebrafish exposure to concentration-dependent groups of BPS, discovering the obviously down-regulated CAT expression [28], which was consistent with this study. It can be seen from Figure 4a that the relative expression of CAT declined markedly with exposure time in the BPS treated group. Meanwhile, the relative expression of GPX and ERβ1 also apparently decreased in the BPZ and BPS treated groups, respectively, which can be seen in Figure 4b,c. In the BPC, BPZ, and BPF treated groups, the CAT and ERβ1 expression showed similar trends. Expression of CAT and ERβ1 declined in the period of 24 h to 48 h and from 72 h to 96 h but slightly increased from 48 h to 72 h (Figure 4a,c). As shown in Figure 4b, the expression of GPX in the BPC, BPF, and BPS treated groups marginally increased from after 48 h to 72 h of exposure and decreased during other exposure time periods. Pikulkaew et al. detected low expression levels of ERβ1 after 8 h post-fertilization, which then increased from 24 h to 48 h post-fertilization in zebrafish [29] A significant decrease of CAT and GPX in the brain and liver of zebrafish was reported by Gyimah et al., which may cause damage to the organs of zebrafish [30].

## 3. Materials and Methods

### 3.1. Chemicals and Materials

Zebrafish (type AB) used in this experiment were all purchased from Shanghai Jiayu Aquarium, with an average body length of 2.5 ± 0.5 cm and an average weight of 0.17 g. After body surface disinfection with 5% sodium chloride solution, the zebrafish were domesticated in tap water which had been dechlorinated after 72 h of aeration. The pH of the test water was 7.74–7.83. The hardness of water is 91–108 mg·L^−1^ (based on CaCO_3_); the concentration of dissolved oxygen was 7.45–7.60 mg·L^−1^; the temperature was controlled at 25 ± 0.5 °C; and the time distribution was a 14:10 h day night cycle. Zebrafish were domesticated in the laboratory for more than 7 days, during which they were fed with commercial feed once a day, and they could not be used in the following experiments until the mortality rate within 7 days was less than 5%.

BPF (98%) was purchased from Shanghai Maclin Biochemical Technology Co., Ltd., Shanghai, China. BPS (99%), BPC (>98.0%), and BPZ (≥98.0%) were purchased from Shanghai Aldin Reagent Co., Ltd., Shanghai, China. Dimethyl sulfoxide (DMSO) was purchased from Shanghai Lingfeng Chemical Reagent Co., Ltd., Shanghai, China. Trizol Reagent was purchased from Thermo Fisher Technology Co., Ltd., Waltham, MA, USA. TaKaRa AMV Kit was purchased from Takara Biomedical Technology (Beijing) Co., Ltd., Beijing, China. dATP, dTTP, dCTP, and dGTP were purchased from Thermo Fisher Technology Co., Ltd., USA. SYBR^®^ Premix Ex Taq^TM^ II (Perfect Real Time) was purchased from Takara Biomedical Technology (Beijing) Co., Ltd., Beijing, China. DNase I was purchased from New England Biolabs Co., Ltd., Ipswich, MA, USA.

Preparation of mother liquor: mother liquor of 10^3^–10^5^ mg·L^−1^ was prepared with DMSO (stored in a refrigerator at 4 °C and kept away from light). DMSO was used as solvent (0.01–0.5%) for all gradient dilution operations.

### 3.2. Experimental Instruments

The durometer was purchased from the Hach Co., Ltd., Loveland, CO, USA. The dissolution oxygen tester was purchased from the Hach Co., Ltd., USA. The electronic analytical balance was purchased from the Shimadsu Co., Ltd., Kyoto, Japan. The ultrasonic curing machine was purchased from the Longjie Ultrasonic Electric Appliance Co., Ltd., Shenzhen, China. The ultra-pure water machine was purchased from the Ultra-Pure Technology Co., Ltd., Sichuan, China. The centrifuge was purchased from Eppendorf Co., Ltd., Hamburg, Germany. The Real-Time PCR System was purchased from Thermo Fisher Technology Co., Ltd., USA. The thermal cycle was purchased from Beijing Dongsheng Innovation Biotechnology Co., Ltd., Beijing, China. The gel electrophoresis and imaging system were purchased from Bio-Rad Laboratories, Inc., Hercules, CA, USA. The Trace UV Nucleic Acid Quantitative System was purchased from Thermo Fisher Technology Co., Ltd., USA.

### 3.3. Experiment Design

#### 3.3.1. Toxicity Test

Zebrafish with sensitive response, normal appearance, and uniform body shape, aged between two and three months old, were selected for the toxicity test. A total length of 2.0–3.0 cm of the zebrafish was selected to be exposed. Glass beakers were selected as the poisoning test container. The volume of each beaker was 3 L, in which 10 zebrafish were placed. There was a blank control, a solvent control (0.01–0.50% dimethyl sulfoxide, DMSO), and a series of bisphenol analogue treatments. For each concentration, three parallel beakers were set up, and all beakers were placed in a water tank with temperature controlled at (25 ± 0.5) °C on a 14:10 light and dark cycle, and the pH value was 7.7–7.8. The experiment period was 96 h, and the test method was static. During the test, there was no feeding or water changes. The test water was dechlorinated tap water that had been aerated for more than 72 h. The number of dead zebrafish was recorded at 6 h, 24 h, 48 h, 72 h, and 96 h, respectively, and the dead fish in the container were removed to avoid affecting the following experiment.

#### 3.3.2. Single Toxicity Test

Before the formal test, preliminary experiments of BPZ, BPC, BPF, and BPS concentrations in a wide range series (0, 1, 1, 10, 100, and 1000 mg·L^−1^) were done. First, 3 L aerated tap water was imported to glass beaker together with reservoir fluid, with no parallel groups. 10 zebrafish were put in each experimental container. During the test, water changes or feeding was permitted, the number of dead fish was then observed and recorded. According to the results of the pre-experiment, 5–7 concentration gradients were set with 3 parallel gradients for each concentration, and blank control experiments were carried out at the same time. The details are as follows: BPF: 1, 2, 4, 6, 8, 10, 12, 14 mg·L^−1^; BPS: 100, 150, 200, 250, 300 mg·L^−1^; BPC: 1, 2, 3, 4, 5 mg·L^−1^; BPZ: 1, 2, 3, 4, 5 mg·L^−1^, and the control group was set as tap water without any drug. The state of the fish was observed at 6 h, 24 h, 48 h, 72 h, and 96 h. After lightly touching the tail of the fish with a glass rod, fish with no reaction were judged to be dead, and the corresponding number of dead fish was recorded. At the end of the experiment, the death rate of the blank control group was less than 10%.

#### 3.3.3. Joint Acute Toxicity Test

Taking the two-element combination as an example, the LC_50_ value of a single compound for 96 h was taken as a toxicity unit, and 6 groups of different experimental concentrations were set using to the 1:1 toxicity ratio with equal logarithmic spacing. The design of the ternary and quaternary combinations was the same. The procedure for the joint acute toxicity test of the combinations was the same as the one conducted for the single compounds. DPS 9.01 software was used to calculate the corresponding LC_50_ value and 95% confidence interval.

#### 3.3.4. Joint Toxicity Assessment Method

At present, there is no unified international standard method for the joint toxicity test of aquatic organisms. In this paper, the additive index method for joint effect of aquatic toxicology by Xiu et al. was used, which was improved from the additive index method of Marking and had been widely used in comprehensive toxicity testing of aquatic toxicology in China [31,32].

Taking the dual combination as an example, after the LC_50_ value of single and combined toxicity was calculated by DPS, S was obtained by Equation (1):

(1) $$S=\frac{A_{m}}{A_{1}}+\frac{B_{m}}{B_{1}}$$

In Equation (1), *A*_1_ and *B*_1_ are LC_50_ values of the single toxicity of poisons A and B, respectively. *A_m_* and *B_m_* are LC_50_ values of each poison in the mixture, respectively. Converting *S* to an additive exponent AI, that is, when *S* ≤ 1, AI = (1/*S*) – 1; When *S* > 1, AI = −*S* + 1. Finally, AI was used to evaluate the combined effect of the toxicants, when AI > 0, the joint toxicity effect is more obvious than the additive effect, that is, the synergistic effect; when AI < 0, the joint toxicity effect is less than the additive effect, that is, antagonism; when AI = 0, the effect of joint toxicity is the addition.

#### 3.3.5. Gene Expression Analysis

Half lethal concentrations of BPZ, BPC, BPS, and BPF were selected in previous experiments, and samples were collected 24 h, 48 h, 72 h, and 96 h after exposure and placed on ice. After homogenization, samples were snap frozen and stored at −80 ℃ until they were assayed. To indicate the possible mechanisms of bisphenol analogue exposure to zebrafish, we also evaluated the gene expression of *β-actin*, *rp17*, CAT, SOD, GPX, ER*α*, and ER*β1* in zebrafish using quantitative real-time polymerase chain reaction (PCR). *β-actin* and *rp17* were selected as internal references, and the other five genes were targets. First, blocks of frozen tissue were quickly ground in liquid nitrogen. 1 mL of Trizol reagent was added after grinding. Total RNA was extracted and isolated using the kit, following the manufacturer’s instructions, meanwhile, cDNA templates were synthesized by the reverse transcription of RNA. For the quantification of PCR results, the Ct value was determined. The *C*t value of *rp17* was lower than *β-actin* and much more easily reproducible. *rp17* was stably expressed across all tested samples. Therefore, *rp17* was chosen for all subsequent sample correction. Primer sequences of the genes are shown in Table 4. The relative gene expression levels were detected by PCR. PCR reaction mixtures (25 μL) contained 12.5 μL of SYBR^®^ Premix Ex Taq^TM^ II, 8.5 μL of nuclease free water, 1 μL of forward primer, 1 μL of reverse primer, and 2 μL of cDNA template. The thermal cycle profile was 95 °C for 2 min, followed by 40 cycles of denaturation for 10 s at 96 °C and 30 s at 60 °C, The PCR was repeated three times for each RNA to generate biological replicates.

### 3.4. Statistical Analysis

DPS 9.01 software was used to analyze the obtained data and the experimental results were expressed as mean ± S.D. The *t*-test method was used to test the statistical difference between the two groups of data. * *p* < 0.05 indicates significant difference. Ct values for each gene of interest were normalized to *rp17* by using the 2^−∆∆Ct^ method [33].

## 4. Conclusions

Bisphenol analogues enter the aquatic environment through different pathways and coexist in water. Multiple substances that act on organisms together may cause different toxic reactions than those of a single substance. In the present study, zebrafish exposure to BPZ, BPS, BPC, and BPF presented different toxic effects. The toxicity of BPZ, BPC, BPF, and BPS decreased sequentially. During the joint toxicity exposure groups, BPF–BPC and BPZ–BPC showed synergistic effects. However, the other joint and multi-joint toxicity exposure groups all exhibited antagonistic effects. Additionally, the estrogen receptor gene (ERβ1) and antioxidant enzyme gene (CAT and GPX) apparently downregulated, indicating that potential risks may be posed by bisphenol analogues. Thus, more studies should be conducted to evaluate the toxicity mechanisms of bisphenol analogues, which would be useful for assessing the health risks associated with bisphenol analogues in the aquatic environment.

## Figures and Tables

**Table 1:** Physicochemical properties of BPC, BPZ, BPF, and BPS.

Analytes	Molecular Weight	CAS	Log Kow	Log BAF	Structures
BPC	256.34	79-97-0	4.74	2.79	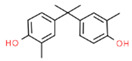
BPZ	268.35	843-55-0	5.48	3.28	_ 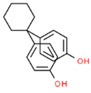 _
BPF	200.23	620-92-8	3.06	1.59	_ 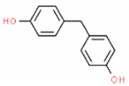 _
BPS	250.27	80-09-1	1.65	0.75	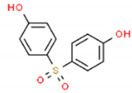

**Table 2:** Combined toxicity of BPZ, BPC, BPF, and BPS to zebrafish after 96 h.

Target	LC_50_/(µM)	S ^1^	AI	Action
BPF–BPS	8.7 × 10^5^(7.6 × 10^5^~9.7 × 10^5^)–2.1 × 10^7^(1.8 × 10^7^~2.3 × 10^7^)	1.07	−0.07	antagonism
BPF–BPZ	8.7 × 10^5^(7.7 × 10^5^~9.6 × 10^5^)–3.7 × 10^5^(3.3 × 10^5^~4.1 × 10^5^)	1.08	−0.08	antagonism
BPF–BPC	7.7 × 10^5^(6.4 × 10^5^~8.5 × 10^5^)–3.4 × 10^5^(2.8 × 10^5^~3.7 × 10^5^)	0.95	0.05	synergism
BPS–BPZ	2.2 × 10^7^(2.0 × 10^7^~2.5 × 10^7^)–4.0 × 10^5^(3.6 × 10^5^~4.5 × 10^5^)	1.15	−0.15	antagonism
BPS–BPC	2.4 × 10^7^(2.2 × 10^7^~2.8 × 10^7^)–4.4 × 10^5^(4.0 × 10^5^~5.2 × 10^5^)	1.25	−0.25	antagonism
BPZ–BPC	3.4 × 10^5^–3.5 × 10^5^	0.99	0.01	synergism

^1^ The sum of the additive effects of biological toxicity.

**Table 3:** Multi-joint acute toxicity of BPZ, BPC, BPF, and BPS on zebrafish after 96 h.

Target	LC_50_/(µM)	S	AI	Action
BPF–BPS–BPZ	6.0 × 10^5^–1.4 × 10^7^–2.6 × 10^5^	1.11	−0.11	antagonism
BPF–BPZ–BPC	6.6 × 10^5^(3.3 × 10^5^~7.6 × 10^5^)–2.8 × 10^5^(1.4 × 10^5^~3.2 × 10^5^)–2.9 × 10^5^(1.5 × 10^5^~3.3 × 10^5^)	1.22	−0.22	antagonism
BPS–BPZ–BPC	1.4 × 10^7^–2.4 × 10^5^–2.5 × 10^5^	1.05	−0.05	antagonism
BPS–BPC–BPF	1.7 × 10^7^–3.1 × 10^5^–7.0 × 10^5^	1.30	−0.30	antagonism
BPF–BPS–BPZ–BPC	4.8 × 10^5^(2.5 × 10^5^~5.6 × 10^5^)–1.1 × 10^7^(5.9 × 10^6^~1.3 × 10^7^)–2.0 × 10^5^(1.1 × 10^5^~2.4 × 10^5^)–2.1 × 10^5^(1.1 × 10^5^~2.4 × 10^5^)	1.18	−0.18	antagonism

**Table 4:** Primer sequences of candidate reference genes.

Gene Symbol	Accession NO.	Primer Sequence(5′-3′)	Product Length (bp)
*β-actin*	AF025305.1	F: CGAGCTGTCTTCCCATCCA R: TCACCAACGTAGCTGTCTTTCTG	86
*rp17*	NM_213213644.2	F: CAGAGGTATCAATGGTGTCAGCCC R: TTCGGAGCATGTTGATGGAGGC	119
CAT	AF170069.1	F: CTCCTGATGTGGCCCGATAC R: TCAGATGCCCGGCCATATTC	126
SOD	BX055516	F: GTCCGCACTTCAACCCTCA R: TCCTCATTGCCACCCTTCC	217
GPX	AW232474	F: AGATGTCATTCCTGCACACG R: AAGGAGAAGCTTCCTCAGCC	94
ER*α*	AF268283	F: CCC ACA GGA CAA GAG GAA GA R: CCT GGT CAT GCA GAG ACA GA	250
ERβ1	AJ414566	F: GGG GAG AGT TCA ACC ACG GAG R: GCT TTC GGA CAC AGG AGG ACG	89

## Data Availability

The authors declare that all data generated or analyzed during this study are included in the published article.

## Sample Availability

Samples of the compounds are available from the authors.
