# Correction: Han et al. Study on the Joint Toxicity of BPZ, BPS, BPC and BPF to Zebrafish. *Molecules* 2021, *26,* 4180

**DOI:** 10.3390/molecules28248098

**Published:** 2023-12-15

**Authors:** Ying Han, Yumeng Fei, Mingxin Wang, Yingang Xue, Hui Chen, Yuxuan Liu

**Affiliations:** 1 School of Environmental & Safety Engineering, Changzhou University, Changzhou 213164, China; 2 Jiangsu Engineering Research Center of Petrochemical Safety and Environmental Protection, Changzhou 213164, China

In the original publication [1], we described the results of the median half lethal concentrations (LC50) of BPZ, BPS, BPC, and BPF to zebrafish for 96 h as 2.57 mg·L^−1^, 155 mg·L^−1^, 2.76 mg·L^−1^, and 8.09 mg·L^−1^, respectively. The joint toxicity effect of BPF–BPC (3.86–1.32 mg·L^−1^) and BPZ–BPC (1.27–1.36 mg·L^−1^) with the same toxic ratio showed a synergistic effect, which may be attributed to enzyme inhibition or induction theory. According to the reviewer’s comments, we transferred mg·L^−1^ to µM. In this process, we used the unit conversion formula, resulting in an unreasonably bigger order of magnitude for the relevant data. We did not notice this mistake before the article was published. So, we need to make corrections for all data described as µM. The corrections of figures and data marked with red colors are as follows.

## 1. Corrected Data in Abstract

“The median half lethal concentrations (LC50) of BPZ, BPS, BPC, and BPF to zebrafish for 96 h were 9.6 µM, 6.2 × 10^2^ µM, 10.8 µM, and 40.4 µM respectively. The joint toxicity effect of BPF–BPC (19.3–5.2 µM) and BPZ–BPC (4.7–5.3 µM) with the same toxic ratio showed a synergistic effect, which may be attributed to enzyme inhibition or induction theory”.

## 2. Corrected Data in Introduction

“Mu et al. conducted an acute toxicity test of four bisphenol analogues on zebrafish embryos, in which the half lethal concentration (LC_50_) value of BPF for 96 h was 97.8 µM [10]”.

“Zhao et al. exposed male zebrafish to 4.0 × 10^−3^ µM and 4.0 × 10^−2^ µM BPS solutions for 28 days and found that the plasma insulin level of male zebrafish was significantly reduced, thus impeding its physiological effect on glucose metabolism, leading to increased liver glucose output and decreased glucose metabolism and storage [13]”.

## 3. Corrected Data of Section 2.1

### 3.1. In the Second Paragraph of Section 2.1

“The LC_50_ of BPZ, BPC, BPF, and BPS was 9.6 µM, 10.8 µM, 40.4 µM, and 6.2 × 10^2^ µM, respectively”.

### 3.2. In the Third Paragraph of Section 2.1

“The LC_50_ values of BPZ in adult zebrafish and embryos after 96 h were 9.8 µM and 10.9 µM, respectively. For BPF and BPS, the LC_50_ values in adult zebrafish were 47.5 µM and 1.4 × 10^3^ µM, and in embryo, they were 37.0 µM and 1.3 × 10^3^ µM [20]”.

## 4. Corrected Data of Section 2.2

### In the First Paragraph of Section 2.2

“As shown in Table 2, the dual combined effect of BPF–BPC (19.3–5.2 µM) with an additive index (AI) value of 0.05 and BPZ–BPC (4.7–5.3 µM) with an AI value of 0.01 showed synergistic effect”.

## 5. Corrected Figure 1

**Figure 1:**
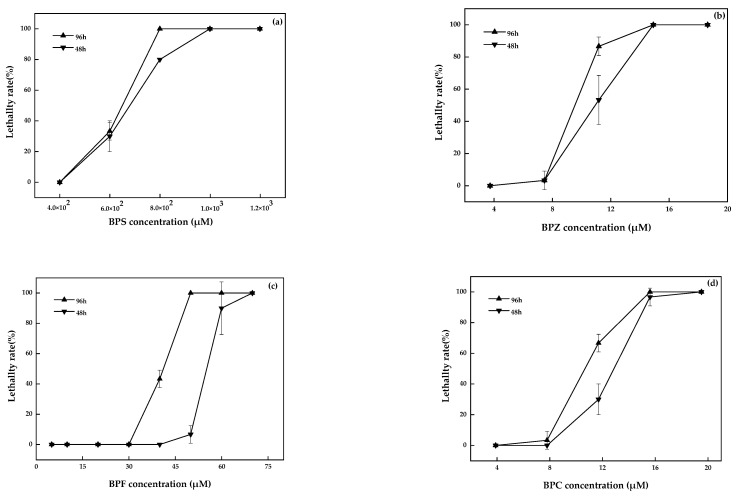
BPS (**a**), BPZ (**b**), BPF (**c**), and BPC (**d**) exposure on zebrafish after 48 h and 96 h (values represent mean ± S.D., sample size: 3).

## 6. Corrected Table 2

**Table 2:** Combined toxicity of BPZ, BPC, BPF, and BPS to zebrafish after 96 h.

Target	LC_50_/(µM)	S ^1^	AI	Action
BPF–BPS	21.7(19.0~24.2)–3.3 × 10^2^	1.07	−0.07	antagonism
	(2.9 × 10^2^~3.7 × 10^2^)			
BPF–BPZ	21.7(19.2~24.0)–5.1	1.08	−0.08	antagonism
	(4.6~5.7)			
BPF–BPC	19.3(15.9~21.3)–5.2	0.95	0.05	synergism
	(4.2~5.7)			
BPS–BPZ	3.6 × 10^2^ (3.2 × 10^2^~4.0 × 10^2^)–5.5	1.15	−0.15	antagonism
	(5.0~6.2)			
BPS–BPC	3.9 × 10^2^ (3.5 × 10^2^~4.5 × 10^2^)–6.8	1.25	−0.25	antagonism
	(6.1~7.9)			
BPZ–BPC	4.7–5.3	0.99	0.01	synergism

^1^ The sum of the additive effects of biological toxicity.

## 7. Corrected Table 3

**Table 3:** Multi-joint acute toxicity of BPZ, BPC, BPF, and BPS on zebrafish after 96 h.

Target	LC_50_/(µM)	S	AI	Action
BPF–BPS–BPZ	15.0–2.3 × 10^2^–3.5	1.11	−0.11	antagonism
BPF–BPZ–BPC	16.4(8.2~18.9)–3.9(1.9~4.5)–4.4(2.2~5.0)	1.22	−0.22	antagonism
BPS–BPZ–BPC	2.2 × 10^2^–3.4–3.8	1.05	−0.05	antagonism
BPS–BPC–BPF	2.7 × 10^2^–4.6–17.4	1.30	−0.30	antagonism
BPF–BPS–BPZ–BPC	11.7(6.1~13.8)–1.8 × 10^2^ (94.2~2.1 × 10^2^)–2.8(1.5~3.3)–3.2(1.6~3.7)	1.18	−0.18	antagonism
